# Use of cuffed flexometallic tube to control profuse airway bleeding during extraction of tracheal metallic foreign body

**DOI:** 10.4103/0019-5049.76569

**Published:** 2011

**Authors:** Ganga Prasad, Gokul Toshniwal

**Affiliations:** Department of Anesthesiology and Intensive Care, All India Institute of Medical Sciences, New Delhi, India; 1 Department of Anesthesiology, Wayne State University/ Detroit Medical Center, Detroit, MI, USA

Sir,

Profuse bleeding through a tracheostoma in a patient with a history of total laryngectomy is a nightmare for any anaesthesiologist. We would like to present an interesting case of profuse airway bleeding in a laryngectomized patient following attempt to extract metallic tongue cleaner (MTC) and its management using a cuffed flexometallic (FM) endotracheal tube.

**Figure 1:**
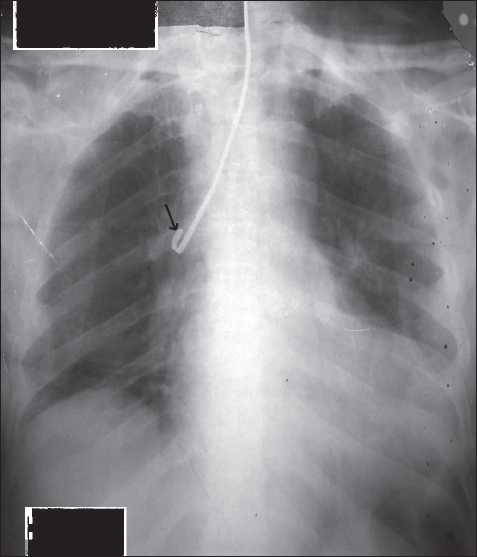
X-ray with metallic tongue cleaner in the airway through tracheostoma. Arrow - Curved distal stuck end

A 78-year-old male presented to us with a protruding MTC through tracheostoma [Figure 1]. The patient was alert, conscious, haemodynamically stable, and maintaining saturation at presentation. There was a history of self-controlled bleeding during initial attempts to extract MTC by the patient. On fiberoptic bronchoscopy, tip of the MTC was found to be stuck to the wall of his right main stem bronchus and there was no obvious bleeding. In an attempt to extract the MTC under flexible fiberoptic bronchoscope guidance led to profuse bleeding. The patient became agitated and his oxygen saturation fell from 96% to 70%. Tracheal suctioning was attempted but there was persistent bleeding and blood flooded his trachea. A 26 FG cuffed FM tube was inserted through the tracheostoma with intermittent suctioning. It was pushed forward with intermittent inflation and deflation of the cuff. At one point, bleeding stopped during inflation of the cuff. The cuff was kept inflated and tube was fixed at that level (9 cm). Position of the tube was confirmed by good and persistent capnograph tracing. Air entry was heard bilaterally with decreased intensity on the left side. Haemodynamic parameters were stable and his oxygen saturation improved to 96–99% on 100% O_2_. Patient was positioned in the left lateral decubitus position for thoracotomy to extract the MTC. The FM tube position was maintained manually, avoiding any displacement. General anaesthesia was induced with sevoflurane and maintained with intravenous (IV) fentanyl, sevoflurane, and 50% nitrous oxide in oxygen and IV vecuronium for muscle relaxation. The MTC was extracted through a right bronchotomy. After closure of the thoracotomy incision, a lumbar epidural (L2-3) catheter was inserted for postoperative pain relief. The FM tube was changed to a tracheostomy tube (size 6.5 mm) and neuromuscular blockade was reversed. Patient was monitored in intensive care unit (ICU) for 24 hours. He was discharged on day 5.

Patients with established tracheostoma are at high risk for aspiration of foreign bodies due to the loss of protective airway reflexes.[1] This can be easily prevented and requires proper education of both the patient and their relatives regarding the care of tracheostomata.[2] These patients may present with a history of prior foreign body aspiration, repeated chest infection, or severe respiratory distress.[3] This was a unique case as patient presented with profuse life threatening bleeding through the tracheostoma and we used cuffed FM tube to control bleeding from inaccessible site in the Tracheo-bronchial tree. The purpose of passing a FM tube was to separate the lungs for oxygenation. The intermittent cuff inflation-deflation technique was useful in identifying the site of bleeding and to control bleeding by tamponade effect of inflated cuff. The alternative way to separate the lung was to use a double lumen tube through tracheostoma.[4] We preferred the FM tube in view of its flexibility and smoothness and easy placement through the tracheostoma. In conclusion, we stress on the usefulness of the cuffed FM tube in scenarios of life threatening bleeding from Tracheo-bronchial tree in a laryngectomized patient. Also, the threshold for open surgical procedure must be low in any situation where the foreign body appears stuck or that previous attempts have led to minor bleeding, so called “Warning bleed”.[5] The anaesthesiologist must be involved early in such cases and collectively plan the extraction of the foreign body in a controlled situation.
